# Primary hypothyroidism and chronotypes in adult women

**DOI:** 10.1186/s13104-022-05934-3

**Published:** 2022-02-14

**Authors:** Marilyn A. Arosemena, Alberto R. Ramos, Erin N. Marcus, Katarzyna A. Slota, Joseph Cheung, Pablo R. Castillo

**Affiliations:** 1 Division of General Internal Medicine, Jackson Memorial Hospital, Miami, FL USA; 2 Department of Neurology, University of Miami, Miami, FL USA; 3 Division of Pulmonary, Allergy and Sleep Medicine, Mayo Clinic, Jacksonville, FL USA; 4 Mayo Clinic School of Graduate Medical Education, Mayo Clinic College of Medicine and Science, Jacksonville, FL USA; 5 Endocrinology, Diabetes, and Metabolism, University of Chicago, Chicago, IL USA; 6 Department of Neurology and Sleep Medicine Program, University of Utah, Salt Lake City, UT USA

**Keywords:** Chronotypes, Circadian rhythm, Hypothyroidism, Sleep–wake phase

## Abstract

**Objective:**

Abnormal thyroid function may disrupt sleep architecture. We aimed to determine the frequency of various chronotypes in women with hypothyroidism. We performed a single-center retrospective study at an ambulatory clinic from January 2013-December 2015. Participants were women with hypothyroidism. Chronotype was determined from the Munich ChronoType Questionnaire. The χ^2^ test was used to compare differences in clinical characteristics and sleep patterns in early and intermediate/late chronotypes. The *t* test was used to compare differences between means.

**Results:**

We evaluated 99 patients (mean [SD], 56 [7] years): calculated chronotype revealed: 56% early, 38% intermediate and 6% late. Analysis with the χ^2^ test showed significant differences between early and intermediate/late calculated chronotypes for sleep latency (*P* = 0.01), light exposure (*P* = 0.009), and no alcohol intake (*P* = 0.001). *t* test showed the following differences in mean (SD) between chronotypes: sleep duration, 7.30 (1.39) hours (early chronotype) and 7.04 (2.06) hours (intermediate/late); body mass index (BMI), 29.4 (7.3) (early) and 31.1 (6.8) (intermediate/late); and TSH level, 2.89 (3.69) mIU/L (early) and 1.69 (1.41) mIU/L (intermediate/late). Early chronotypes were frequent in women with hypothyroidism. Light exposure and BMI may influence chronotypes in patients with hypothyroidism; findings are consistent with healthier behaviors in patients who tend toward morningness.

**Supplementary Information:**

The online version contains supplementary material available at 10.1186/s13104-022-05934-3.

## Introduction

Circadian rhythms, which are genetically determined intrinsic systems that influence the sleep–wake cycle, are critical for health and optimal organ function. The circadian clock controls physiology at many levels, partly through the hypothalamic-pituitary–peripheral organ axes. An excess or a deficit of hormones can disrupt sleep architecture [1].

Chronotypes are defined by the timing of sleep onset, wakefulness and behavior (early vs. late activities). Chronotypes are classified as early (morningness) to describe those who go to bed early and wake up early; late (eveningness) to describe those who go to bed late and wake up late; and intermediate. Patients with hypothyroidism have an increased risk for sleep disturbances [2, 3]. However, a paucity of information exists on the relationship between chronotypes and thyroid disease. Hypothyroidism is more common in females, and young females tend to have an early chronotype that can change to a late chronotype with older age [4–6].

This study aimed to determine the chronotypes in women with hypothyroidism and the association of the chronotypes with demographic and clinical characteristics. In recognition of the impact of aging, we hypothesized that patients with hypothyroidism would more likely have late chronotype.

## Main text

### Methods

#### Population

We evaluated 99 patients at an urban ambulatory center who were identified through electronic health records (EHR). Patients were women, age 18 to 65, who had clinical hypothyroidism and were treated with levothyroxine from January 1, 2013, through December 31, 2015. Only women were selected because of higher hypothyroidism prevalence (female to male ratio 4–6:1) [7]. Exclusion criteria: shift workers, diagnosis of sleep apnea in the preceding 2 months and primary hypersomnia.

One of the authors tried to contact 450 patients by phone. Participants were asked questions from the Munich ChronoType Questionnaire (MCTQ). Of the 450 patients, 351 were excluded: 203 did not answer the phone; 12 declined to answer; 5 had sleep apnea; 6 shift workers; 3 were pregnant; 4 had a mental disability preventing from answering questions; 116 were > 65 years; 1 had died; 1 had a TSH of 118 mIU/L.

#### Outcome

We used the MCTQ to quantitatively assess participant’s sleep. MCTQ focuses on asking about work schedule, workdays, sleep timing preceding workdays, work-free days, and use of an alarm clock [1, 4–6].

Chronotype was defined by the timing of sleep onset and wakefulness. Self-defined (reported) chronotype was defined as the reported time when individuals preferred to perform certain activities: early, intermediate or late [8]. All analyses were made on workdays and work-free days. Data regarding sleep onset, duration, time in bed, sleep loss, light exposure, and chronotype were obtained. (Additional file 1: Table S1). Midsleep was defined as the midpoint between sleep onset and awakening. Calculated chronotype was computed from midsleep on work free days only; chronotype was categorized as early (< 3 AM), intermediate (from 3–5 AM), and late (> 5 AM). Chronotypes according to midsleep could be calculated only in patients without alarm use on weekends.

#### Exposures and covariates

The MCTQ included demographic questions and the patient’s sleep schedule on workdays/work-free days, which included continuous variables such as time of going to bed, sleep latency, sleep end, sleep inertia, work schedule, and light exposure. Body mass index (BMI) was determined from the EHR and categorized as normal (18.5–25), overweight (25.1–30), or obese (> 30). Categorical variables were alarm clock use (yes/no); use of stimulants such as coffee (cups daily, further differentiated into 0, 1–3 [light use], and > 3 [heavy use]), current tobacco use (yes if > 1 cigarette daily), alcohol (yes if > 1 drink weekly), or drugs (yes/no); and sleep medications (self-defined). Calculated variables were obtained with MCTQ formulas.

In addition, we obtained clinical information from EHR: TSH (Thyroid stimulating hormone) (euthyroid, 0.27–4.20 mIU/L; hyperthyroid < 0.27 mIU/L; hypothyroid > 4.20 mIU/L); levothyroxine dose; presence of diabetes; and hemoglobin A1c (HbA1c). A diagnosis of diabetes was determined from EHR per the ADA guidelines, HbA1c level was recorded only for patients with diabetes (A1c ≤ 7% was considered well-controlled diabetes; HbA1c > 7%, uncontrolled).

After answering the questionnaire, participants classified themselves into a chronotype: early, intermediate, or late.

#### Statistical analysis

Descriptive statistics included age, BMI, workdays per week, sleep schedule, tobacco use, alcohol use, sleep medication use, and reported chronotype. The χ2 test was used to determine differences in the proportion of clinical characteristics (categorical variables) and the chronotype categories of early and intermediate/late chronotypes. Intermediate and late chronotypes were grouped together as the number of patients with late chronotype was small. Calculated chronotype was chosen instead of reported as both correlated very well and calculated chronotype is an objective measure. The t test was used to compare differences between means. For univariate analyses, we used Astatsa and Excel with an α level of 0.05. To examine the determinants of calculated chronotype in our sample, we fitted logistic regression models to independently test the associations between each demographic, behavioral, and sleep risk factor. Two regression models were fitted for the outcome of calculated intermediate/late chronotype: (1) first model adjusted for age, BMI, and TSH; (2) second model adjusted for those 3 variables and for the use of alcohol and coffee. The variables included in the models were associated with the outcome in univariate analysis or were significantly different across the calculated chronotypes. SAS 9.4 software was used for the logistic regression models.

### Results

99 patients met the inclusion criteria and answered the MCTQ. The mean (SD) age of the patients was 56.5 (7.0) years. The BMI category was obese for 40.4%. Mean TSH was 1.43 mIU/L, 73.7% patients were biochemically euthyroid (Table 1).

**Table 1:** Features of Female Patients With Hypothyroidism (N = 99)

Feature	No	%
Age, y		
22–59	54	54.5
60–65	45	45.4
BMI		
18.5–25 (Normal)	17	17.2
25.1–30 (Overweight)	42	42.4
> 30 (Obese)	40	40.4
Thyrotropin, mIU/L		
< 0.27	14	14.1
0.27–4.20	73	73.7
> 4.20	12	12.1
Diabetes		
Yes	23	23.2
No	76	76.8
Hemoglobin A_1c_ (n = 23)		
≤ 7%	10	43.5
> 7%	13	56.6
Levothyroxine dose, mcg		
< 50	3	3
50–100	62	62.6
> 100	34	34.3
Workdays per week, no.		
0–2	15	15.2
3–5	41	41.4
> 5	43	43.4
Cigarette smoking		
Yes	6	6.1
No	93	93.9
Alcohol intake		
Yes	17	17.2
No	82	82.8
Coffee intake, no. of cups daily		
0	16	16.2
1–3	53	53.5
> 3	30	30.3
Sleep medication (weekly)		
Yes	26	26.3
No	73	73.7
Chronotype (reported)		
Early	63	63.6
Intermediate	24	24.2
Late	12	12.1

	Median	Interquartile ranges
Workdays: time of going to bed	10:45 PM	(9PM–11PM)
Workdays: sleep latency, min	40	(5–90)
Workdays: sleep end	6:30 AM	(5:30AM–7AM)
Workdays: sleep inertia, min	5	(3–15)
Workdays: light exposure, min	30	(10–120)
Work-free days: time of going to bed	10:30 PM	(8PM–10:30PM)
Work-free days: sleep latency, min	30	(5–90)
Work-free days: sleep end	7:05 AM	(6AM–8AM)
Work-free days: sleep inertia, min	5	(3–15)
Work-free days: light exposure, min	30	(10–120)

*BMI* body mass index (calculated as weight in kilograms divided by height in meters squared)

MCTQ responses regarding work-days and work-free days bed time, sleep latency, sleep end and inertia are seen in Table 1.

Most patients reported working at home (53.5%). Most patients did not smoke (93.9%), or drink alcohol (82.8%), 53.5% drank 1–3 cups of coffee daily, 26.3% used sleep medication at least once weekly for insomnia. The most commonly used medications were melatonin, zolpidem, diphenhydramine, and benzodiazepines. (Table 1).

Analysis of daily schedules showed that for the largest percentages of patients, sleep onset occurred from 9:01 to 11 PM on workdays (46.5%) and from 11:01 PM to 1 AM on work-free days (43.4%); sleep duration was 7–8 h on workdays (40.4%) and on work-free days (38.4%); and 8 to 10 h was spent in bed on workdays (56.6%) and on work-free days (53.5%). For the largest percentages of patients, midsleep occurred < 3 AM on workdays (59.6%) but between 3 and 5 AM on work-free days (47.5%). For the largest percentages of patients, average weekly sleep duration was < 7 h (38.4%), weekly sleep loss was < 1 h (83.8%), and average weekly light exposure was > 40 min (47.5%).

Reported chronotypes were early for 64% of patients, intermediate for 24.2%, and late for 12.1%. Midsleep occurred later on work-free days than on workdays for the largest percentages of patients, and midsleep was later on workdays and on work-free days for patients < 40 years than for patients 40 years or older. Sleep duration was longer on work-free days than on workdays, and patients > 60 years had shorter sleep duration than those < 40 years.

Calculated chronotype was derived for 81 patients who did not report use of an alarm on weekends. Of the 81 patients, 56% had early, 38% had intermediate and 6% had late chronotype. Analysis with the χ2 test showed significant differences between the calculated early vs. intermediate vs. late chronotype: Sleep latency (10^3^ min) was associated with the intermediate chronotype (P = 0.02); no alcohol intake (P = 0.004) and greater average weekly light exposure were associated with the early chronotype (P = 0.007). Once intermediate/late chronotypes was grouped, similar results were found. Sleep latency was associated with the intermediate/late chronotype (P = 0.01); no alcohol intake (P = 0.001) and greater average weekly light exposure were associated with the early chronotype (P = 0.009) (Table 2).

**Table 2:** Chronotypes and sleep patterns of female patients with hypothyroidism (N = 81)

Feature	Patients with chronotype, %				Patients with chronotype, %		
	Early	Intermediate	Late	*P* value	Early	Intermediate/late	*P* value
Age, y				0.82			0.23
22–59	30.8	18.5	3.7		28.9	41.7	
60–65	24.7	19.7	2.5		71.1	58.3	
BMI				0.12			0.052
18.5–30 (Ideal and overweight)	39.5	18.5	3.7		71.1	50	
> 30 (Obese)	16	19.7	2.5		28.9	50	
Thyrotropin, mIU/L				0.25			0.06
< 4.20	44.4	35.8	6.2		80	94.4	
≥ 4.20	11.1	2.5	0		20	5.6	
Diabetes				0.64			0.81
Yes	13.6	9.9	0		24.4	22.2	
No	41.9	28.4	6.2		75.6	77.8	
Hemoglobin A_1c_, %				0.07			0.09
≤ 7	10.5	26.3	0		18	57.1	
> 7	47.3	15.8	0		81.8	8.3	
Levothyroxine dose, mcg				0.77			0.09
< 100	28.4	17.3	3.7		73.3	55.5	
≥ 100	27.2	20.9	2.5		26.7	44.4	
Sleep latency, min				0.02			0.01
< 10	19.8	3.7	1.2		35.5	11.1	
≥ 10	35.8	34.6	4.9		64.4	88.9	
Total time in bed, h				0.45			0.18
< 8	23.5	11.1	1.2		42.2	27.8	
≥ 8	32	27.2	4.9		57.8	69.4	
Average weekly sleep duration, h				0.67			0.88
< 7	22.2	17.3	1.2		40	41.7	
≥ 7	33.3	20.9	4.9		60	58.3	
Weekly sleep loss, h				0.14			0.47
< 1	44.4	34.6	3.7		80	86.1	
≥ 1	11.1	3.7	2.5		20	13.9	
Average weekly light exposure, min				0.007			0.009
< 40	20.9	23.5	6.2		45.5	66.7	
≥ 40	34.6	14.8	0		62.2	33.3	
Cigarette smoking				0.78			0.78
Yes	3.7	3.7	0		6.7	8.3	
No	51.9	34.6	6.2		93.3	91.7	
Alcohol intake				0.004			0.001
Yes	2.5	11.1	2.5		4.4	30.6	
No	53	27.2	3.7		95.6	69.4	
Coffee intake				0.49			0.22
No	9.9	3.7	0		17.8	8.3	
Yes	45.6	34.6	6.2		82.2	91.7	
Sleep medication				0.47			0.26
Yes	12.3	13.6	1.2		22.2	33.3	
No	43.2	24.7	4.9		77.8	66.7	
Sleep duration, h				0.63			0.96
< 7	23.5	17.3	1.2		42.2	41.7	
≥ 7	32	20.9	4.9		57.8	58.3	

BMI, body mass index (calculated as weight in kilograms divided by height in meters squared)

Use of the t test showed the following differences in the mean (SD) between calculated chronotypes: sleep duration, 7.30 (1.39) hours (early chronotype) and 7.03 (1.41) hours (intermediate) and 7.01 (1.55) (late chronotype); age, 58.0 (5.7) years (early), 56.8 (7.4) years (intermediate) and 55.7 (6.9); BMI, 29.4 (7.3) (early), 30.2 (7.1) (intermediate) and 30.1 (5.4) (late); and TSH, 2.89 (3.69) (early), 2.3 (2.9) (intermediate) and 2.4 (2.2) (late) mIU/L. Linear regression of TSH and midsleep revealed r^2^ = 0.07 (p = 0.01) (Additional file 1: Fig. S1).

A lower BMI and higher TSH increased the odds of having an early chronotype. Alcohol consumption increased the odds for an intermediate/late chronotype (Table 3).

**Table 3:** Determinants of intermediate/late chronotype for women with hypothyroidism (N = 81)

Feature	Odds ratio (95% CI)	
	Model 1	Model 2
Age in years	0.9 (0.8–1.0)	0.9 (0.8–1.0)
BMI	1.07 (0.9–1.1)	1.08 (0.9–1.1)
Thyrotropin level	0.8 (0.65–0.99)	0.79 (0.63–1.00)
Alcohol use (yes vs no)	–	11.06 (1.9–61.7)
Coffee use (yes vs no)	–	1.79 (0.35–8.9)

BMI, body mass index (calculated as weight in kilograms divided by height in meters squared)

## Discussion

To our knowledge, this is the first study to assess chronotypes in hypothyroidism. Even though patients with hypothyroidism have more sleep disturbances, there is a scarcity of data on the relationship of circadian patterns and thyroid disease [9, 10].

The key finding in our study is that patients with hypothyroidism are more likely to have an early chronotype. A possible explanation why higher TSH correlates with early chronotype may be related to higher TSH secretion in the early evening and peak secretion during the early part of the night [11]. Higher TSH seems to shift the circadian clock towards morningness. In addition, we found that higher BMI is associated with increased TSH.

In studies of chronotypes in healthy individuals and in patients with depression and inflammatory bowel disease, late chronotype is a marker of circadian misalignment (eg, social jet lag, sleep debt, and inconsistent meal timing) [12, 13]. To our knowledge there are no comparable studies of chronotype in hypothyroidism. It is well known that, compared with people who have an early chronotype, people who have a late chronotype are more likely to have unhealthy behaviors and higher risk for obesity. In a prior study, women with a late chronotype had larger weight gains and higher BMI than women who had an early chronotype [14]. Similar observations have been made with patients with hypothyroidism and an intermediate/late chronotype.

In the present study, most patients had a normal TSH, which indicates euthyroid state; this may explain why most patients have an early chronotype [14]. However, in patients with normal thyroid glands, circulating TSH have a clear daily rhythm that is disrupted in patients with hypothyroidism [15]. This disruption is unlikely to be mitigated by standard doses of levothyroxine; thus, it is possible that continued variance in thyroid hormone during the day may lead to a change in chronotype.

Chronotypes are influenced by the environment, society, and medical conditions. Brain cortical thickness varies between people with different chronotypes [16], and an early chronotype is associated modestly with a lower risk of depression, but the mechanism is unclear [17] and the implications are partially uncertain.

Early chronotypes were more frequent in this sample of women with hypothyroidism. Light exposure, BMI, and lack of thyroid hormone rhythmicity may influence chronotypes in women who have hypothyroidism. Our findings are consistent with the observation in other studies that healthier behaviors are more common among patients who have a tendency toward morningness.

## Limitations

Limitations include a small sample size of female patients from an urban sample, which limits the implications to other populations. The patients were at a low-income clinic, mostly uninsured whose literacy level may be low. A subject with a TSH higher than 20 was excluded (outlier for linear regression analysis) but results with/without outlier were the same. Many patients reported adequate sleep onset, latency, and duration, yet some reported waking up in the middle of the night, which may have interfered with sleep quality. A considerable proportion of patients were obese and responses may have been confounded by undiagnosed sleep apnea. The present study did not exclude patients on sleep medications. Age-related changes in sleep propensity may have contributed to awakening early in the morning, with phase-advanced in older patients; nevertheless, our patients awakened early regardless of age after we controlled for age in our statistical models [18].

Sunlight and latitude are important in the sleep–wake cycle, chronotype is affected by geography, and people living in higher latitudes have a tendency toward eveningness [19]. Our study was conducted in Florida, where the latitude is lower than most US cities, so more hours of sunlight may have influenced chronotype in our sample.

## Supplementary Information

**Additional file 1:** **Table S1.** Munich ChronoType Questionnaire Variables. **Figure S1.** Linear regression between TSH and midsleep

## Data Availability

Due to patient privacy we cannot share PHI. All data is shown in Table 1. We will be able to provide only de-identified data of our patients to confirm/reproduce analysis. These could be requested by sending an email to the corresponding author Dr. Arosemena.

## Abbreviations

EHR: Electronic health record
MCTQ: Munich ChronoType Questionnaire
BMI: Body mass index (calculated as weight in kilograms divided by height in meters squared)
TSH: Thyroid stimulating hormone
HbA_1c_: Hemoglobin A_1c_
