# Supplementary material for: Primary hypothyroidism and chronotypes in adult women
Source: BMC Res Notes. 2022 Feb 14;15:52. doi: 10.1186/s13104-022-05934-3 (PMC8842526; doi:10.1186/s13104-022-05934-3)
Supplement: Supplementary file 1 — Additional file 1: Table S1. Munich ChronoType Questionnaire Variables. Figure S1. Linear regression between TSH and midsleep [file 13104_2022_5934_MOESM1_ESM.docx]

**Supplementary information**

**Supplementary Table 1.** Munich ChronoType Questionnaire Variables

| **Variable** | **Statement** | **Format** | **Workdays** | | **Work-free days** | |
| --- | --- | --- | --- | --- | --- | --- |
|  |  |  | **Abbreviation** | **Computation** | **Abbreviation** | **Computation** |
| Local time of going to bed | I go to bed at __ o’clock | hh:mm | BT_w_ | NA | BT_f_ | NA |
| Local time of preparing to sleep | I actually get ready to fall asleep at __ o’clock | hh:mm | SPrep_w_ | NA | SPrep_f_ | NA |
| Sleep latency | I need __ minutes to fall asleep | mm | SLat_w_ | NA | SLat_f_ | NA |
| Sleep end | I wake up at __ o’clock | hh:mm | SE_w_ | NA | SE_f_ | NA |
| Alarm clock use | With an alarm clock | yes/no | Alarm_w_ | NA | Alarm_f_ | NA |
| Sleep inertia | After __ minutes, I get up | mm | Sl_w_ | NA | Sl_f_ | NA |
| No. of workdays and work-free days per week | I have a regular work schedule and work __ days per week | No. | WD | NA | FD | 7-WD |
| Light exposure | On average, I spend the following amount of time outdoors in daylight (without a roof above my head) | hh:mm | LE_w_ | NA | LE_f_ | NA |
| Sleep onset | NA | hh:mm | SO_w_ | SPrep_w_ + SLat_w_ | SO_f_ | SPrep_f_ + SLat_f_ |
| Local time of getting out of bed | NA | hh:mm | GU_w_ | SE_w_ + Sl_w_ | GU_f_ | SE_f_ + Sl_f_ |
| Sleep duration | NA | hh:mm | SD_w_ | SE_w_ – SO_w_ | SD_f_ | SE_f_ – SO_f_ |
| Total time in bed | NA | hh:mm | TBT_w_ | GU_w_ – BT_w_ | TBT_f_ | GU_f_ – BT_f_ |
| Midsleep | NA | hh:mm | MSW | SO_w_ + SD_w_/2 | MSF | SO_f_ + SD_f_/2 |

Abbreviations: hh, hour; mm, minute; NA, not applicable.

**Supplementary Figure 1.** Linear regression between TSH and midsleep


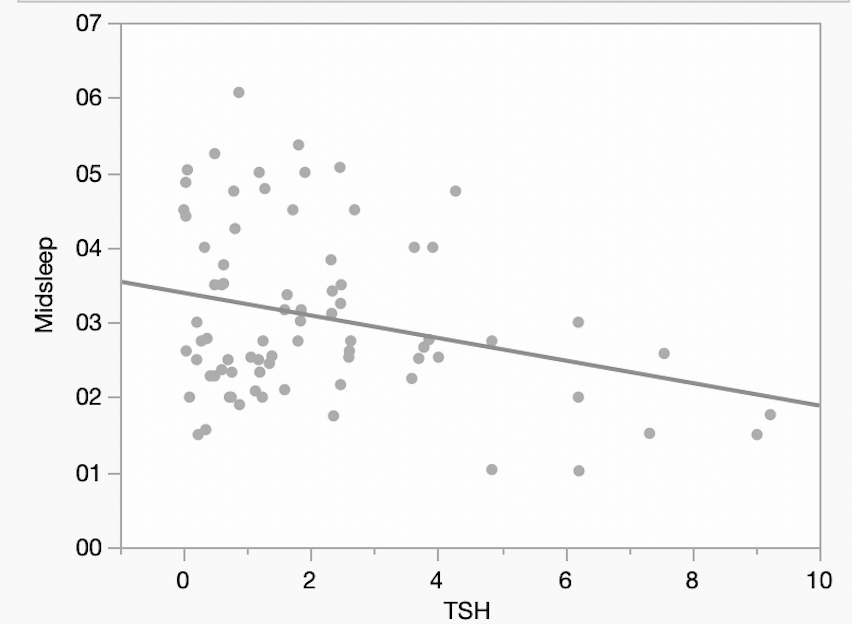


n=80*

r^2^= 0.07

p=0.01

*One outlier subject with a TSH of 20 was excluded from the analysis. Analysis with and without the outlier showed the same correlation.
